# Nitrogen rates and plant density interactions enhance radiation interception, yield, and nitrogen use efficiencies of maize

**DOI:** 10.3389/fpls.2022.974714

**Published:** 2022-09-23

**Authors:** Peiyu Tian, Jiamin Liu, Yanan Zhao, Yufang Huang, Yanhao Lian, Yang Wang, Youliang Ye

**Affiliations:** Agricultural Green Development Engineering Technology Research Center, College of Resources and Environment, Henan Agricultural University, Zhengzhou, China

**Keywords:** canopy N distribution, light gradient, N use efficiency, maize, individual, population

## Abstract

The contributions of the different leaf layers to maize yields identified as middle leaf > lower leaf > upper leaf, where the vertical photosynthetically active radiation (PAR) in the canopy gradually decreases. We hypothesized that the allocation of more PAR and nitrogen (N) to the highest contributing leaves will would be beneficial for higher yields and N use efficiencies. The N application rate and plant density effectively regulated the canopy light and N distribution. We evaluated the interactive effects of N rate and plant density on the agronomic and ecophysiological characteristics of leaves at different orientations in a 2019/2020 field experiment. In this study, an N application rate of 180 kg ha^–1^ coupled with a plant density of 82,500 plants ha^–1^ achieved the highest yield and N recovery efficiency (NRE). In contrast to the traditional farming practices in northern China, the density was increased and N rate was reduced. Densification from 52,500 to 82,500 plants ha^–1^ increased the population leaf area index (LAI) by 37.1% and total photosynthetically active radiation (TPAR) by 29.2%; however, excessive density (from 82,500 to 97,500 plants ha^–1^) drastically reduced the proportion of TPAR by 28.0% in the lower leaves. With increased density, the leaf areas and angles of the upper leaves decreased much more than those of the other leaves, which allowed the middle and lower leaves to access more light, which manifested a smaller extinction coefficient for light (*K*_L_). A high yield (>1,000 kg ha^–1^) of maize could be achieved simultaneously with higher NRE; however, it was negatively correlated with internal N use efficiency (IE_N_). Higher N concentrations and lower total performance index (PI_*total*_) in the lower leaves may be an important rationale for the reduction of IE_N_ in high-yielding maize. Additionally, decreased N rate without yield reduction under higher densities was primarily attributed to the more uniform vertical N distribution [a smaller extinction coefficient for N (*K*_N_)]. These results suggest that the N fertilizer rate can be moderately reduced without a reduction in maize yield under high plant densities in northern China.

## Introduction

Maize (*Zea mays* L.) is a major cereal crop worldwide and a staple food in developing countries (Nedi et al., 2016). Increasing grain yields with reduced inputs is an important goal for sustainable agriculture. Maize grain yield is positively related to the number of kernels per unit land area. Kernels arise from well-developed and pollinated female florets, that are borne by spikelet meristems derived from the inflorescence meristem (Ning et al., 2021). For high-yield maize breeding, kernel number per ear is one of the key breeding targets (Zhou et al., 2015). In cultivation, there are two approaches for increasing the kernel number per unit area: (i) increasing the number of grains per unit area by increasing the planting density and (ii) increasing the number of florets under the same planting density to increase the number of grains per ear (Yan et al., 2018). The former needs to overcome the adverse effects of dense planting conditions on floret development, whereas the latter requires improvements in the activities of inflorescence and florets, which can increase the number of fertile florets (Cheng et al., 1983).

In China, most farmers produce maize under conditions of low plant density and high fertilizer input. In practice, low plant densities (5.0–6.0 plants m^–2^) and the over-application of Nitrogen (N) fertilizer (approximately 250 kg N ha^–1^) restrict yields and N use efficiencies (Jin et al., 2012). In contrast, grain yields as high as 15.2 t ha^–1^ have been achieved in some studies under high plant densities (9.0–10.5 plants m^–2^) and N inputs (approximately 750 kg N ha^–1)^ in northern China (Li et al., 2001). Therefore, appropriate plant densities combined with optimal N management are likely to increase grain yields and N use efficiency (Yan et al., 2016).

N is the most limiting nutrient in maize production; thus, it is required in larger amounts than to other nutrients (Correndo et al., 2021). During the silking stage, maize exhibits a reduced vegetative plant N accumulation rate and increased N remobilization rate (from vegetative organs to grains). Thus, deficiencies in N accumulation prior to anthesis affect kernel numbers of reductions in carbon assimilation (Uhart and Andrade, 1995). Limited N decreases starch deposition in the maize endosperm, which resulting in a decline in kernel weight, primarily through its influence on the synthesis of sucrose synthase, hexokinase, and pyrophosphate-linked phosphofructokinase (Singletary and Below, 1990). Consequently, to ensure high yields, N fertilizer overdosing relative to the actual needs of plants is practiced by most farmers in China (Xu et al., 2017). However, the overapplication of N fertilizer enhances N losses through runoff, denitrification, leaching, and volatilization (Li et al., 2018; Hou et al., 2021). Furthermore, high N fertilizer inputs lead to luxury absorption, and increased the risks of lodging, diseases, and pests (Kruczek, 2003).

Plant density influences the light environment of plant canopies (Luo et al., 2018). This is because the leaf area and angle of a single plant are reduced with increasing plant density, whereas the upper leaf area is increased, which inhibits light interception of the middle and lower leaves (Mina, 2013; Al-Naggar and Atta, 2017). Ecological studies have demonstrated that light harvesting is of paramount importance for plants growing in light competitive environments such as dense stands (Hou et al., 2019). In the upper canopy layer, the allocation of N to the photosynthetic apparatus of the leaves increases with higher radiation, which results in enhanced N use efficiencies. In contrast, N allocations in the mid- and lower-canopy layers decrease with higher plant densities, resulting in a reduction of light-saturated photosynthetic rates and photosynthetic N use efficiencies in leaves (Yao et al., 2016). An appropriate maize plant density provides a good microecological environment that is beneficial for individual plants as well as coordination between groups (Shi et al., 2016). From this perspective, medium plant densities are optimal as they facilitate the highly efficient utilization of light, the superior spatial allocation of leaf N to the photosynthetic apparatus, and high use efficacy of photosynthetic N in leaves within the canopy (Yao et al., 2016). This strategy has been employed to increase the yields of wheat (Liu et al., 2021; Zheng et al., 2021), cotton (Li et al., 2017), rice (Hou et al., 2019), and oilseed rape (Labra et al., 2020).

It is noteworthy that the accumulation of N in individual plants decreases with higher plant densities (Wang et al., 2020), because crowding stress reduces the capacity of plants to absorb soil N (Yan et al., 2017). Thus, more N fertilizer is required as planting density increases. Except for a few reports of super high-yielding crops, most studies have concluded that increased plant densities with reduced N inputs can improve their efficacy while maintaining grain yields (Dong et al., 2019; Du et al., 2021). This raises the question of why high N inputs under high plant densities do not attain high yields. Furthermore, the optimal plant density optimizes the distribution of N in maize to enhance its efficiency. In this study, we compared the agronomic and photochemical characteristics of different canopy layers at various N input rates and plant densities. Furthermore, we clarified the effects of light and leaf N matching on maize yields and N use efficiencies.

## Materials and methods

### Site description

**FIGURE 1:**
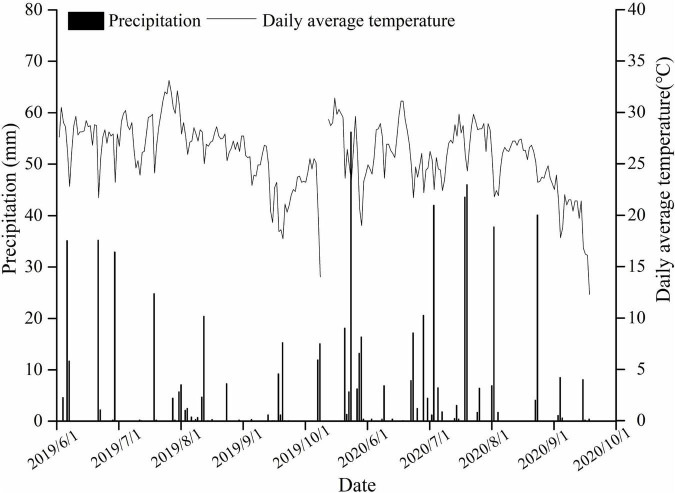
Temperature and precipitation measurements during the maize-growing season.

Field experiments were conducted during the maize growing seasons (June–October 2019, 2020) in Yuzhou County (34°27′N 113°34′E), Henan Province, Central China. During the maize-growing seasons, the total precipitation and mean temperature were 321.2 mm and 18.0°C, respectively, in 2019, and 467.0 mm and 24.4°C, respectively, in 2020 (Figure 1). Prior to the experiments, soil samples were extracted from the upper 20-cm layer for chemical analyses. The soil type was fluvoaquic soil (pH 8.2), with an organic matter content of 16.3 g kg^–1^, total N of 1.04 g kg^–1^, available P of 20.0 mg kg^–1^, available K of 113.7 mg kg^–1^, available Zn of 1.15 mg kg^–1^, and bulk density of 1.25 g cm^–3^.

### Experimental design and management

The cultivar Beiqing 340 was used for the experiments during the two growing seasons. This maize cultivar has been widely cultivated by Henan farmers because of its high yields and adaptability. Beiqing 340 is a compact plant type, with an average plant height of 279 cm and 19 leaves. It has sturdy stems, well-developed aerial roots, and good lodging resistance (ensuring that maize will not lodge under high-density planting in this study).

The experiment was laid out using a split-plot design with three replicates, with plant densities assigned to the primary plots, whereas the N input rates were set up in subplots. These included four plant densities (52,500, 67,500, 82,500, and 97,500 plants ha^–1^, abbreviated as *D*_525_, *D*_675_, *D*_825_, and *D*_975_, respectively) and three N input rates (0, 180, and 360 kg ha^–1^, abbreviated as *N*_0_, *N*_180_, and *N*_360_, respectively). The dimensions of each plot were 4 m × 10 m and seeds were mechanically sown on June 03. Urea served as the source of N, which was applied in two splits, with 50% at the basal stage and 50% at the 10-leaf stage (45 days after sowing). Phosphorus (90 kg [P_2_O_5_] ha^–1^) in the form of calcium superphosphate, potassium (90 kg [K_2_O] ha^–1^) in the form of potassium chloride and zinc (5 kg [Zn] ha^–1^) in the form of zinc sulfate were applied as the basal dose. Basal fertilizer was applied to the ground following manual broadcasting, whereas N topdressing was applied by means of side-dressing. Nicosulfuron and atrazine were applied at the three-leaf stage to control weeds, whereas thiophanate-methyl and lambda-cyhalothrin were applied at the eight-leaf stage to prevent diseases and insects.

### Sampling and measurements

#### Leaf position

**FIGURE 2:**
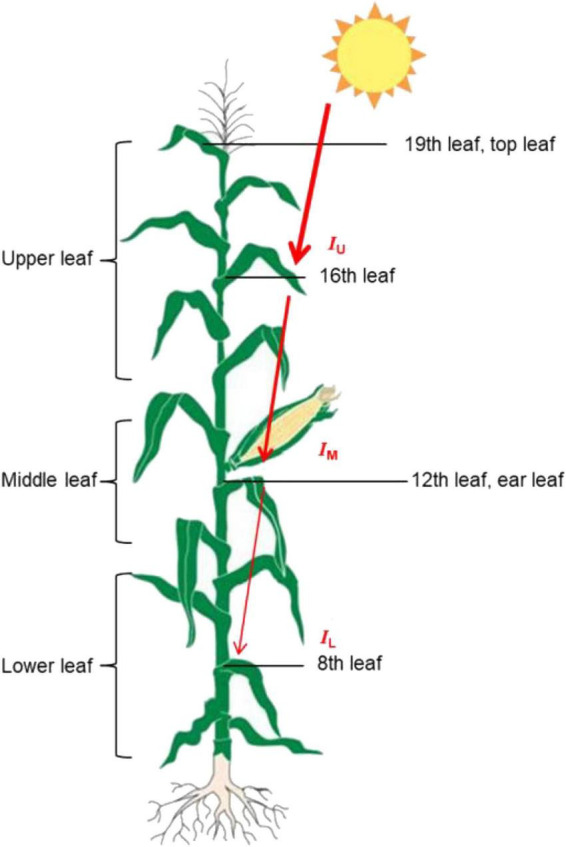
A schematic model depicting the leaf position and incident light of maize. The *I*_U_, and *I*_o_, *I*_M_, and *I*_L_ are PAR values on a horizontal level at upper, middle and lower leaf layers. The thickness of the red arrows represent the light density that is intercepted by different leaf positions.

The ear leaf, a leaf above the ear leaf, and a leaf below the ear leaf were defined as the middle leaf (M leaf). The remaining leaves above the middle leaf were referred to as the upper leaf (U leaf; the six leaves above the middle leaf), while the remaining leaves below the M leaf were referred to as the lower leaf (L leaf; there are only five to six leaves below the middle leaf at the silking stage, because some of the early emerging lower leaves would have died). Beiqing 340 had a total of 19 leave. We measured the agronomic (except leaf area) and physiological indicators of the 16th, 12th, and 8th leaves (from the base to the top, the base leaf was the 1st leaf, the ear leaf was the 12th leaf, and the top leaf was the 19th leaf), which represent the upper, middle, and lower leaves, respectively (Figure 2).

#### Leaf agronomic and physiological characteristic measurements

At the silking stage (75 days after sowing), the PAR was measured using a field scout external light sensor meter (3415FX and 366816 quantum light 6 sensor bar, Spectrum, CA, United States) from 11:00 to 12:30 pm, during sunlight hours. The sensor bar was horizontally placed into the different leaf positions facing upward to measure the PAR. The TPAR was calculated as follows:

TPAR (μmol s^–1^) = PAR (μmol ⋅ m^–2^ ⋅ s^–1^) × leaf area (m^2^).

The area of each living leaf was measured at the silking stage (R1). The leaf area was determined using a leaf area meter (YMJ-A; Zhejiang Top Yunnong Technology Co., Ltd., China), and the U, M, and L leaf areas were the sum of the areas of all leaves at that leaf position. The leaf area index (LAI) is the surface area of leaves per unit of ground. The leaf angle was measured using a protractor, and the leaf angle was defined as the acute angle value between the leaf and stem.

At the silking stage, the leaves at different leaf positions of five representative plants in each plot were selected as physiological test materials in the morning (10:00–1200 am). The leaf disk samples were homogenized in 5 mL of an 80% acetone solution added to 0.01 g of CaCO_3_, and then centrifuged at 2,000 × *g* for 10 min at 10°C. The supernatant was collected, and the final volume of the extract was 25 mL using 80% acetone. The absorbance (A) of the extracts was determined at 663 and 645 nm using a spectrophotometer, and an estimate of the chlorophyll content a [Chl a = 12.72 × *A*_663_ − 2.59 × *A*_645_], chlorophyll b [Chl b = 22.88 × *A*_645_ − 4.67 × *A*_663_], chlorophyll (a + b) [Chl (a + b) = 20.29 × *A*_6452_ + 8.05 × *A*_663_] was obtained according to Li (2000).

The remaining leaf disk samples were used to measure relative leaf conductivity (EC), and 40 mL of distilled water was added to the samples in a clean beaker. Subsequently, the conductivity *R*_0_ was quantified using a conductivity meter (DDSJ 308, Shanghai), which was placed in a beaker sealed with plastic wrap and soaked for 5–6 h, after which the conductivity R_1_ was measured. Next, the samples were placed in a water bath, boiled for 30 min, and removed. After cooling to room temperature, conductivity *R*_2_ was measured again. The relative conductivity EC was calculated as EC = (*R*_1_ − *R*_0_)/(*R*_2_ − *R*_0_), according to Fu et al. (2020).

To obtain the dry weight of the leaves, the leaf samples were dried at 105°C for 30 min, and then at 70°C to a constant weight, ground to pass through a 1-mm mesh screen, and then digested with H_2_SO_4_ and H_2_O_2_. The total N concentration of the digested samples was determined using an automated continuous flow analyzer (Seal, Norderstedt, Germany).

#### Leaf chlorophyll fluorescence measurements

The chlorophyll a fluorescence was measured for 10 s via a Plant Efficiency Analyzer (PEA; Hansatech Ltd., King’s Lynn, Norfolk, United Kingdom) with an excitation light intensity of 3.3 mmol m^–2^ s^–1^, which emits light that is centered at a 650 nm wavelength with an intensity of 3,000 μmol photons m^–2^ s^–1^. The fluorescence emission was detected using a high-performance PIN-photodiode with an optical design and filtering that ensured a maximal response to longer wavelength fluorescence signals. These measurements were taken from the leaves (30 min dark-adapted) at the maize silking stage, in which 15 leaves at different orientations were measured for each plot.

Each transient was analyzed according to the JIP-test (Appenroth et al., 2001), by utilizing the original data: *F*_0_ (minimum fluorescence, when all PSII reaction centers were open), *F*_m_ (maximum fluorescence, when all PSII reaction centers were closed), *M*_0_ (approximated initial slope of the fluorescence transient), and *V*_j_ and *V*_i_ (fluorescence intensities at 2 and 60 ms, respectively). The following equations were used for the quantification of PSII behavior, referring to time zero: (1) the specific energy fluxes per cross section (CS) for absorption (ABS/CS_0_), trapping (TR_0_/CS_0_), electron transport (ET_0_/CS_0_), dissipation (DI_0_/CS_0_), and PSI electron acceptation (RE_0_/CS_0_):

ABS/CS_0_ = *F*_0_;

TR_0/_CS_0_ = (1–*F*_0_/*F*_M_) × *F*_0_;

ET_0/_CS_0_ = (1–*F*_0_/*F*_M_) × (1-*V*_j_) × *F*_0_;

DI_0/_CS_0_ = ABS/CS_0_–TR_0_/CS_0_;

RE_0/_CS_0_ = (ET_0_/CS_0_) × (1–*V*_*j*_) × (1–*V*_*i*_),

(2) The number of active PSII reaction centers per excited cross-section (RC/CS_0_)

RC_/_CS_0_ = (*F*_*M*_ – *F*_0_) × (*V*_*j*_/*M*_0_)

(3) Total performance index (PI_*total*_) of the photosynthetic apparatus on an absorption basis:

PI_*total*_ = (RC/ABS) × [(TR_0_/ABS)/(1–TR_0_/ABS)] × [(ET_0_/TR_0_)/(1–ET_0_/TR_0_)] × [(RE_0_/ET_0_]/(1–RE_0_/ET_0_)].

### Calculations and statistical analysis

The relationship between canopy light distribution and canopy structure may be described by an exponential function (Gallagher and Biscoe, 1978) as:

*I*_*M*_ = *I*_*U*_ . e ^(–^**^K^*^L(U^*^–^*^M)^* ^.^ *^F^*^(U–*M*))^; *I*_L_ = *I*_*M*_ . e ^(–^**^K^*^L(M^*^–^*^L)^* ^.^ *^F^*^(M–*L*))^

where *F*_(_*_*U*–*M*_*_)_ and *F*_(_*_*M*–L_*_)_ are the cumulative areas of green leaves per unit ground from the upper to middle leaf layer and from the middle to lower leaf layer, respectively; *I*_*U*_, *I*_*M*_, and *I*_L_ are PAR values on a horizontal level in the upper, middle, and lower leaf layers, respectively; and *K*_*L(U*–*M)*_ and *K*_*L(U*–*M)*_ are the light extinction coefficients from the upper to middle leaf layer and from the middle to lower leaf layer, respectively. A smaller *K*_L_ value indicates a more uniform light distribution in the canopy.

The N gradient is described by the following with an exponential function (Anten et al., 1995) as:

*N*_*M*_ = (*N*_*U*_–*N*_*b*_) . e^(–^*^K^*^N(U–^*^M)^* ^.^ *^F^* ^(U–*M*))^ + *N*_*b*_; *N*_L_ = (*N*_*M*_–*N*_*b*_) . e^(–^*^K^*^N(M–^*^L)^* ^.^ *^F^*^(M–*L*))^ + *N*_*b*_

where *N*_*U*_, *N*_*M*_, and *N*_L_ are the leaf N (g N m^–2^) of the upper, middle, and lower leaf layers, respectively; *K*_*N(U–M)*_ and *K*_*N(M–L)*_ are the extinction coefficients for effective leaf N from the upper to middle leaf layer and from the middle to lower leaf layer, respectively; and *N*_*b*_ is the base value of leaf N for photosynthesis, which may be regarded as representing the non-photosynthetic N content. The value of *N*_*b*_ is 0.3 g N m^–2^ leaf (Yin and van Laar, 2005).

The formula for calculating the absorption and utilization efficiency parameters of the N fertilizer:

TNA [kg ha^–1^] = plant N concentration [kg kg^–1^] × plant dry matter [kg ha^–1^]

IE_N_ [kg kg^–1^] = grain yield [kg ha^–1^]/total plant N uptake [kg ha^–1^]

NRE [%] = (TNA of N applied - TNA of N omitted) [kg ha^–1^]/N applied [kg ha^–1^] × 100 [%]

where TNA is the total N accumulation, IE_N_ is the internal N-use efficiency, and NRE is the N recovery efficiency.

The data from both seasons were statistically evaluated using an analysis of variance (*ANOVA*) to compare the differences between the various treatments, and the means were separated using the least significant difference (LSD) test at a significance level of 0.05. Variance analyses of N rate, plant density, and their interactive effects were performed using the Statistical Software Package for Social Science (SPSS, version 19.0). Figures were generated using Origin Pro 9.0.

## Results

### Grain yields

**FIGURE 3:**
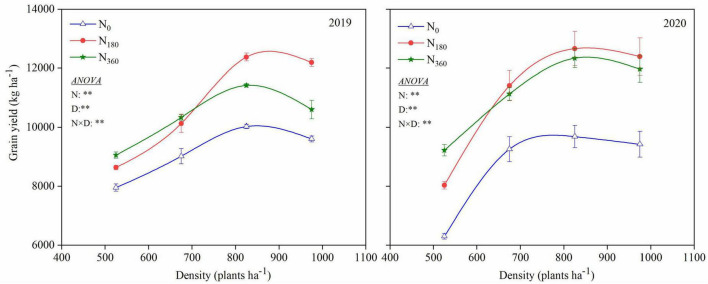
Influence of N rate and plant density on the grain yield.

Two-way ANOVAs revealed that the grain yield was markedly affected by the N rate (N), plant density (D) and their interaction effect (N × D) in 2019 and 2020 (Figure 3). Compared with the *N*_0_ input, the grain yields were remarkedly increased under the *N*_180_ input at averaged across planting densities, averaging 1681.0 and 2454.2 kg ha^–1^ for the 2 years at increase rates of 17.8 and 28.2%, respectively. Compared with *N*_180_, the grain yield with the *N*_360_ input under the *D*_525_ treatment was substantially increased. No notable change in grain yield was observed under the *D*_675_ treatment; however, it was markedly reduced by *D*_975_ treatment.

Higher plant densities resulted in greater grain yields, with the largest being under the *D*_825_ treatment at 2722.7 and 3708.2 kg ha^–1^, with increase rates of 31.8 and 47.2%, respectively, recorded for the 2 years. Higher plant densities led to relatively higher grain yields with a lower N application rate, and vice versa. The *N*_180_ input combined with *D*_825_ treatment for 2 years was the optimal combination for the best yields among all treatments.

### Leaf agronomic traits at different leaf positions

**FIGURE 4:**
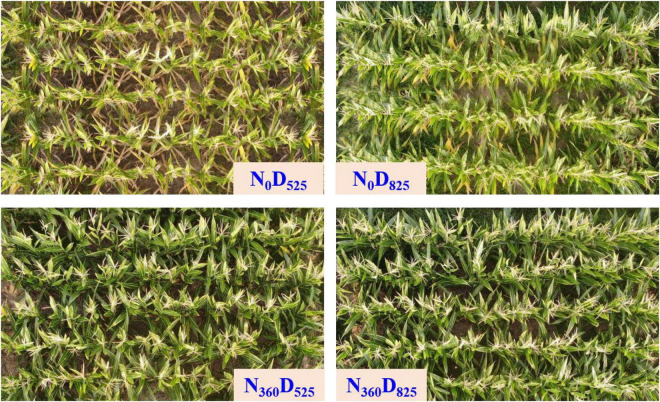
Top view of different nitrogen input and plant density treatments on leaf development at the tasseling stage.

LAI, leaf area, and leaf angle at different leaf orientations were affected by N rates and plant density; however, there were no strong interaction between them (Figure 4 and Table 1). Compared with the *N*_0_ input averaged across planting densities, the LAI of the *N*_180_ input was increased by from 19.4 to 23.5% in 2019 and from 14.3 to 22.0% in 2020. With continued increase in the N fertilizer rate to 360 kg ha^–1^, the LAI decreased. Higher plant density also substantially significantly influenced leaf development. Compared with the *D*_525_ treatment averaged across the N treatments, the LAI under *D*_825_ treatment increased by 26.9% in 2019 and 47.3% in 2020. With a further increase in planting density, compared to the *D*_825_ treatment, the LAI under the *D*_975_ treatment increased by 13.9% in 2019 and 12.1% in 2020.

**TABLE 1:** Influence of N rate and plant density on the leaf area index (LAI), leaf area, and angle at different leaf positions.

Density	N rate	LAI	Leaf area (m^2^/plant)			Leaf angle (°)		
				
			Upper	Middle	Lower	Upper	Middle	Lower
**2019**								
*D*_525_	*N*_0_	3.6 ± 0.2 b	0.18 ± 0.00 b	0.22 ± 0.02 b	0.29 ± 0.03 ab	16.3 ± 1.5 b	24.3 ± 3.2 b	24.0 ± 2.3 b
	*N*_180_	4.3 ± 0.3 a	0.21 ± 0.00 a	0.28 ± 0.02 a	0.34 ± 0.05 a	19.7 ± 0.6 a	26.7 ± 3.1 a	27.3 ± 1.0 a
	*N*_360_	4.0 ± 0.1 ab	0.19 ± 0.01 b	0.28 ± 0.02 a	0.29 ± 0.01 b	18.3 ± 1.5 ab	28.7 ± 0.6 a	27.3 ± 1.0 a
*D*_675_	*N*_0_	4.5 ± 0.2 b	0.17 ± 0.01 b	0.21 ± 0.02 b	0.29 ± 0.03 ab	15.0 ± 0.6 b	23.7 ± 2.9 b	23.3 ± 1.0 b
	*N*_180_	5.5 ± 0.5 a	0.21 ± 0.00 a	0.27 ± 0.03 a	0.33 ± 0.05 a	17.7 ± 1.2 a	26.3 ± 2.0 ab	25.3 ± 2.0 a
	*N*_360_	5.0 ± 0.2 ab	0.19 ± 0.01 ab	0.28 ± 0.02 a	0.28 ± 0.01 b	15.7 ± 1.5 ab	27.3 ± 1.7 a	25.0 ± 1.0 a
*D*_825_	*N*_0_	4.5 ± 0.1 b	0.14 ± 0.01 b	0.18 ± 0.01 b	0.22 ± 0.00 b	13.7 ± 2.1 a	21.7 ± 3.2 b	22.0 ± 0.6 b
	*N*_180_	5.5 ± 0.3 a	0.15 ± 0.02 ab	0.23 ± 0.01 a	0.28 ± 0.04 a	15.3 ± 1.2 a	26.0 ± 3.3 a	25.0 ± 0.6 a
	*N*_360_	5.1 ± 0.2 a	0.16 ± 0.01 a	0.19 ± 0.03 ab	0.27 ± 0.02 ab	14.7 ± 1.5 a	25.8 ± 2.3 a	24.0 ± 0.6 a
*D*_975_	*N*_0_	5.1 ± 0.0 b	0.12 ± 0.01 b	0.18 ± 0.01 b	0.22 ± 0.00 b	9.3 ± 0.6 b	17.7 ± 2.3 b	20.3 ± 1.2 a
	*N*_180_	6.3 ± 0.5 a	0.14 ± 0.02 ab	0.22 ± 0.00 a	0.28 ± 0.03 a	13.3 ± 1.7 a	22.7 ± 0.4 a	22.0 ± 0.6 a
	*N*_360_	5.8 ± 0.4 a	0.15 ± 0.01 a	0.19 ± 0.03 ab	0.26 ± 0.03 ab	12.0 ± 1.7 a	22.5 ± 1.0 a	21.3 ± 1.7 a
*N*		**	**	**	**	**	**	*
*D*		**	**	**	**	**	**	**
*N* × *D*		ns	ns	ns	ns	ns	ns	ns
**2020**								
*D*_525_	*N*_0_	3.5 ± 0.1 b	0.18 ± 0.01 b	0.23 ± 0.02 b	0.26 ± 0.01 b	15.0 ± 1.0 b	24.0 ± 1.0 b	23.7 ± 1.5 b
	*N*_180_	4.0 ± 0.2 a	0.23 ± 0.00 a	0.25 ± 0.01 a	0.29 ± 0.02 a	17.7 ± 1.5 a	27.3 ± 1.2 a	27.3 ± 1.2 a
	*N*_360_	3.7 ± 0.2 ab	0.21 ± 0.02 ab	0.24 ± 0.00 ab	0.27 ± 0.01 ab	15.7 ± 0.6 ab	27.3 ± 1.5 a	26.3 ± 1.5 ab
D_675_	*N*_0_	4.2 ± 0.1 b	0.17 ± 0.02 b	0.20 ± 0.02 b	0.23 ± 0.01 b	12.5 ± 0.5 b	18.0 ± 2.6 b	16.3 ± 1.2 b
	*N*_180_	5.0 ± 0.5 a	0.22 ± 0.02 a	0.25 ± 0.01 a	0.28 ± 0.02 a	15.0 ± 1.0 a	25.5 ± 2.0 a	21.3 ± 2.4 ab
	*N*_360_	4.5 ± 0.2 ab	0.21 ± 0.04 ab	0.23 ± 0.00 ab	0.25 ± 0.01 ab	13.7 ± 1.2 ab	23.3 ± 1.5 a	24.5 ± 2.3 a
*D*_825_	*N*_0_	5.0 ± 0.1 c	0.16 ± 0.00 b	0.22 ± 0.00 b	0.22 ± 0.01 b	9.3 ± 0.6 b	23.3 ± 1.2 b	17.7 ± 0.6 b
	*N*_180_	6.1 ± 0.2 a	0.20 ± 0.02 a	0.25 ± 0.01 a	0.29 ± 0.01 a	13.3 ± 1.5 a	25.3 ± 0.6 a	22.7 ± 2.5 a
	*N*_360_	5.4 ± 0.2 b	0.17 ± 0.02 ab	0.24 ± 0.00 ab	0.26 ± 0.02 a	12.0 ± 1.0 a	25.0 ± 1.0 ab	22.7 ± 1.5 a
*D*_975_	*N*_0_	5.8 ± 0.1 b	0.16 ± 0.00 b	0.20 ± 0.00 b	0.21 ± 0.01 b	7.5 ± 0.9 b	17.3 ± 2.5 b	19.3 ± 0.5 b
	*N*_180_	6.8 ± 0.2 a	0.19 ± 0.02 a	0.25 ± 0.01 a	0.25 ± 0.01 a	10.7 ± 1.1 a	23.5 ± 0.9 a	21.7 ± 1.6 a
	*N*_360_	5.9 ± 0.2 b	0.16 ± 0.01 ab	0.22 ± 0.00 b	0.25 ± 0.02 a	10.3 ± 2.1 a	23.1 ± 2.2 a	20.8 ± 1.2 ab
*N*		**	**	**	**	**	**	**
*D*		**	**	**	**	**	**	**
*N* × *D*		ns	ns	ns	ns	ns	ns	ns

* and ** indicate that the yield components were significantly influenced by N rate, plant density, and their interactions at 0.05 and 0.01 levels, and ns indicates ‘not significant’. Different lowercase letters following the values in the same column indicate a significant difference at the same density level at P < 0.05.

The area of the lower leaf was higher than that of the middle or U leaf. Similar to the LAI being affected by N application rate, the leaf areas at different orientations were enhanced with the N rate, and the maximum value appeared under *N*_180_ input. The leaf area per plant was decreased substantially at higher plant densities. Compared with the *D*_525_ treatment averaged across N treatments, the U leaf area under the *D*_825_ treatment showed the greatest decreases of 22.4% (2019) and 14.5% (2020), followed by the L leaf and M leaf. Comparison with the *D*_825_ treatment, the leaf area at different leaf positions continued to decrease.

Akin to the leaf area, the leaf angle also affected the amount of intercepted light. The leaf angle of the U leaf was the lowest, followed by those of the M, and L leaves. At the same planting density, the leaf angles under various N inputs were notably higher than those under *N*_0_ input; however, no obvious differences were observed between them. Leaf angles also decreased at higher plant densities. Compared with the *D*_525_ treatment averaged across N treatments, the U leaf angle under the *D*_825_ treatment decreased by 19.5% (2019) and 28.5% (2020), the M leaf angle decreased by 7.8% (2019) and 6.4% (2020), and the L leaf angle decreased by 9.7% (2019) and 18.4% (2020). In comparison with the *D*_825_ treatment, the leaf angles at different leaf positions continued to decrease.

### Intercepted photosynthetic radiation at different leaf positions

**FIGURE 5:**
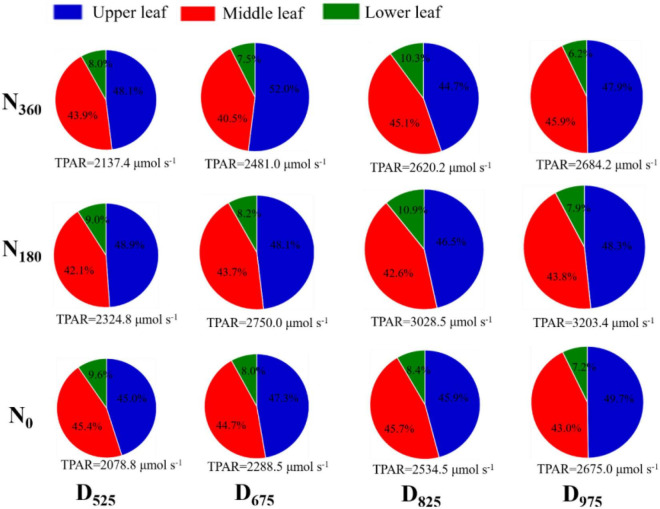
Influence of N rate and plant density on the photosynthetically active radiation (PAR) at different leaf positions in 2019. The size of pie diagram represents the TPAR of different leaf layers; the percentage of each section represents the TPAR ratio for the upper, middle, and lower leaf layers.

The area of the pie chart represents the TPAR intercepted by different leaf layers (Figure 5 and Supplementary Figure 1). In 2019, the average TPAR of the U leaves was 22.4% higher than that of the M leaves, and 3.67 times higher than that of the L leaves across all treatments. In 2020, the average TPAR of the U leaves was 37.5% higher than that of the M leaves, and 4.19 times higher than that of the L leaves at across all treatments. For the same leaf positions, TPAR increased with higher planting densities and higher N application rates (except for *N*_360_ treatments). For U leaves, the average TPAR ratio (TPAR of any leaf layer/TPAR of the whole population) increased with higher plant densities, ranging from 46.3 to 48.9% (2019) and from 45.7 to 49.1% (2020) across N treatments. However, the TPAR ratio for L leaves decreased with higher plant densities, ranging from 10.0 to 7.1% (2019) and from 9.5 to 7.1% (2020). The change in the TPAR value of M leaves among the different densities was very small, ranging from 4.4 to 4.5% (2019) and from 4.3 to 4.5% (2020).

### Leaf photosynthetic characteristics at different leaf positions

**FIGURE 6:**
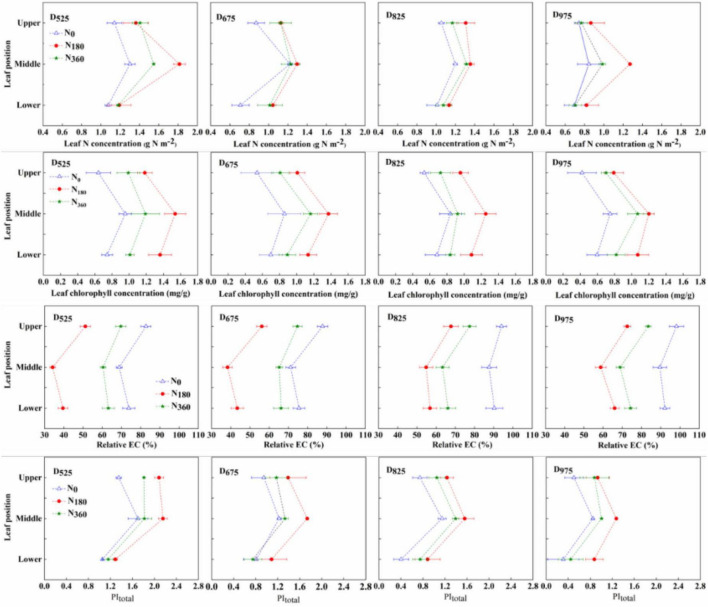
Influence of N rate and plant density on the leaf N concentration, chlorophyll concentration, relative EC, and PI_total_ at different leaf positions in 2019.

Under the same treatments, the photosynthesis-related parameters (N concentration, chlorophyll concentration, fluorescence efficiency, and senescence process) of leaves at different positions varied (Figure 6 and Supplementary Figure 2). The M position leaf possessed the highest leaf N, chlorophyll, and PI_*total*_, and the lowest relative EC, followed by the U and L leaves.

At the same plant density, the specific leaf N (SLN) concentration, chlorophyll concentration, and PI_*total*_ of the *N*_180_ inputs were always better than those of the *N*_360_ inputs, with the worst being the N_0_ input. The relative EC parameter, the opposite trend was observed for the relative EC parameter between different N input rates. At the same N input rates, SLN concentration, chlorophyll concentration and PI_*total*_ decreased with higher plant densities, but the highest value was always observed with *N*_180_. In 2019, compared with the *D*_525_ treatment, the SLN concentration under the *D*_825_ treatment decreased by 11.6% (U leaf, 6.6%; M leaf, 17.0%; and L leaf, 9.7%), leaf chlorophyll concentration decreased by 18.4% (U leaf, 16.2%; M leaf, 18.0%; and L leaf, 21.3%), leaf PI_*total*_ was decreased by 36.4% (U leaf, 41.9%; M leaf, 27.7%; and L leaf, 42.2%), and leaf relative EC increased by 21.0% (U leaf, 20.8%; M leaf, 25.8%; and L leaf, 17.4%). Further increasing planting density, compared with the *D*_825_ treatment, the SLN concentration under the *D*_975_ treatment decreased by 27.5% (U leaf, 31.0%; M leaf, 19.8%; and L leaf, 32.7%), leaf chlorophyll concentration decreased by 5.4% (U leaf, 4.6%; M leaf, 0.2%; and L leaf, 13.6%), leaf PI_*total*_ decreased by 22.9% (U leaf, 20.0%; M leaf, 23.8%; and L leaf, 23.7%), and the leaf relative EC increased by 6.9% (U leaf, 9.0%; M leaf, 5.6%; and L leaf, 6.3%). A similar trend was observed for 2020.

Specific leaf fluxes are presented in Table 2. The ABS/CS_0_, TR_0_/CS_0_, and ET_0_/CS_0_ of the M leaves were significantly higher than those of the U and L leaves during the two growing seasons. At the same plant density, the ABS/CS_0_, DI_0_/CS_0_, TR_0_/CS_0_, and ET_0_/CS_0_ of the *N*_180_ at any leaf position were the highest, followed by the *N*_360_, with the worst being the *N*_0_ input. It is worth noting that under the same N rates, ABS/CS_0_, DI_0_/CS_0_, TR_0_/CS_0_, and ET_0_/CS_0_ at any leaf position decreased with higher plant densities. In 2019, compared with the *D*_525_ treatment, the ABS/CS_0_ under the *D*_825_ treatment was decreased by 3.3% (U leaf), 6.2% (M leaf), and 4.7% (L leaf) at the different leaf positions. The ABS/CS_0_ of *D*_975_ was decreased by 0.7% (U leaf), 0.6% (M leaf), and 0.7% (L leaf) in comparison with *D*_825_. In 2020, compared with the *D*_525_ treatment, the ABS/CS_0_ under the *D*_825_ treatment was decreased by 11.7% (U leaf), 12.6% (M leaf), and 10.5% (L leaf) at the different leaf positions. The ABS/CS_0_ of *D*_975_ decreased by 1.0% (U leaf), 0.9% (M leaf), and 1.0% (L leaf) in comparison with *D*_825_. Similarly, with an increased in planting density (from *D*_525_ to *D*_825_), the rate of decrease of TR_0_/CS_0_ and ET_0_/CS_0_ in M leaves was higher than that in other leaf layers.

**TABLE 2:** Influence of N rate and plant density on the leaf specific energy fluxes per cross section (CS) at different leaf positions.

Density	N rate	Upper				Middle				Lower			
		
		ABS/CS_0_	DI_0_/CS_0_	TR_0_/CS_0_	ET_0_/CS_0_	ABS/CS_0_	DI_0_/CS_0_	TR_0_/CS_0_	ET_0_/CS_0_	ABS/CS_0_	DI_0_/CS_0_	TR_0_/CS_0_	ET_0_/CS_0_
**2019**													
*D*_525_	*N*_0_	366.9 ± 3.1 c	70.0 ± 2.2 b	296.9 ± 1.9 c	178.0 ± 2.2 c	400.0 ± 2.0 b	75.2 ± 2.2 b	324.8 ± 1.2 c	187.2 ± 2.2 a	360.3 ± 4.1 c	68.0 ± 2.1 b	292.4 ± 2.0 c	174.6 ± 1.3 b
	*N*_180_	381.1 ± 2.5 a	74.2 ± 2.2 a	306.9 ± 1.3 a	182.8 ± 1.2 a	419.5 ± 2.3 a	82.3 ± 3.2 a	337.3 ± 1.1 a	188.3 ± 1.9 a	377.5 ± 2.7 a	71.2 ± 1.0 a	306.3 ± 1.3 a	186.6 ± 2.2 a
	*N*_360_	375.1 ± 3.1 b	70.8 ± 1.9 b	304.3 ± 2.2 b	180.0 ± 1.1 b	402.3 ± 3.0 b	76.0 ± 3.1 b	326.2 ± 1.1 b	187.4 ± 1.3 a	368.3 ± 2.5 b	70.6 ± 1.2 ab	297.7 ± 1.3 b	175.3 ± 2.0 b
*D*_675_	*N*_0_	364.5 ± 2.9 c	68.3 ± 2.4 b	296.2 ± 1.7 c	177.3 ± 2.5 c	397.6 ± 2.2 b	74.5 ± 2.5 b	323.1 ± 1.5 c	185.5 ± 2.4 a	358.9 ± 3.6 c	67.3 ± 1.8 b	291.7 ± 1.8 c	173.9 ± 1.7 b
	*N*_180_	379.1 ± 3.5 a	73.5 ± 2.5 a	305.6 ± 1.9 a	185.5 ± 1.5 a	417.5 ± 1.8 a	81.0 ± 1.7 a	336.6 ± 1.4 a	187.6 ± 1.7 a	374.9 ± 1.9 a	69.9 ± 0.5 a	305.0 ± 2.0 c	185.3 ± 2.8 a
	*N*_360_	372.1 ± 2.6 b	69.5 ± 1.5 b	302.6 ± 2.4 b	182.3 ± 1.4 b	399.3 ± 2.5 b	74.3 ± 3.2 b	324.9 ± 1.8 b	186.1 ± 2.0 a	364.9 ± 2.4 b	68.9 ± 1.2 ab	296.0 ± 1.4 b	173.6 ± 2.2 b
*D*_825_	*N*_0_	356.6 ± 3.1 b	66.9 ± 2.0 b	289.7 ± 3.1 b	167.3 ± 1.1 c	376.5 ± 2.0c	73.7 ± 1.2 c	302.8 ± 2.2 c	172.9 ± 1.3 b	345.2 ± 3.1 b	62.3 ± 0.9 b	283.0 ± 2.2 b	171.9 ± 1.1 b
	*N*_180_	366.4 ± 2.7 a	69.7 ± 1.3 a	296.8 ± 1.2 a	176.2 ± 2.2 a	388.9 ± 1.9 a	74.6 ± 1.4 a	314.3 ± 1.9 a	180.1 ± 2.2 a	355.9 ± 3.1 a	66.2 ± 1.0 a	289.7 ± 3.1 a	173.6 ± 1.1 a
	*N*_360_	363.0 ± 1.3 a	68.6 ± 1.1 a	294.4 ± 2.1 a	169.4 ± 1.9 b	380.7 ± 2.2 b	74.2 ± 1.2 b	306.5 ± 2.0 b	174.0 ± 3.1 b	352.7 ± 2.2 a	64.6 ± 1.4 a	288.1 ± 1.2 a	171.9 ± 2.2 b
*D*_975_	*N*_0_	354.2 ± 3.9 c	66.2 ± 1.8 b	288.0 ± 3.2 c	166.6 ± 1.4 b	374.1 ± 2.2 c	72.0 ± 1.5 b	302.1 ± 2.5 c	171.2 ± 1.4 b	343.8 ± 2.7 c	61.6 ± 0.7 c	282.3 ± 2.0 b	169.2 ± 1.5 ab
	*N*_180_	364.4 ± 2.2 a	68.4 ± 2.0 a	296.1 ± 1.4 a	174.9 ± 2.8 a	386.9 ± 1.7 a	73.9 ± 1.5 a	313.0 ± 1.5 a	179.4 ± 2.0 a	353.3 ± 3.0 a	64.9 ± 0.5 a	288.4 ± 2.5 a	172.3 ± 1.6 a
	*N*_360_	360.0 ± 1.5 b	66.9 ± 1.3 ab	293.1 ± 1.6 b	167.7 ± 2.1 b	377.7 ± 1.7 b	72.9 ± 1.6 ab	304.8 ± 2.2 b	172.7 ± 2.5 b	349.3 ± 1.5 b	62.9 ± 0.4 b	286.4 ± 1.5 a	170.2 ± 2.2 b
*N*		**	**	**	**	**	**	**	**	**	**	**	**
*D*		**	**	**	**	**	**	**	**	**	**	**	**
*N* × *D*		ns	*	Ns	**	**	**	ns	**	**	ns	**	**
**2020**													
*D*_525_	*N*_0_	346.5 ± 1.4 c	76.3 ± 2.2 c	270.2 ± 2.8 b	159.9 ± 1.3 c	371.0 ± 1.1 c	63.7 ± 1.2 c	307.4 ± 1.2 c	173.8 ± 0.7 b	345.2 ± 1.4 c	62.6 ± 2.3 b	282.6 ± 2.0 c	154.8 ± 1.1 c
	*N*_180_	394.4 ± 2.5 a	88.7 ± 1.2 a	305.7 ± 1.4 a	178.5 ± 1.2 a	407.8 ± 1.4 a	70.9 ± 1.3 a	336.9 ± 1.3 a	181.7 ± 3.6 a	363.1 ± 2.5 a	68.3 ± 3.2 a	294.7 ± 2.3 a	178.5 ± 0.4 a
	*N*_360_	357.2 ± 1.2 b	86.7 ± 1.3 b	270.5 ± 2.3 b	165.5 ± 2.3 b	386.7 ± 2.3 b	66.0 ± 1.4 b	320.7 ± 2.2 b	174.8 ± 2.3 b	355.5 ± 3.2 b	62.7 ± 1.2 b	292.9 ± 1.9 b	158.4 ± 2.2 b
*D*_675_	*N*_0_	343.2 ± 3.2 c	74.7 ± 2.7 b	268.5 ± 1.5 b	158.3 ± 0.8 c	367.7 ± 2.0 c	62.0 ± 0.6 c	305.7 ± 0.4 c	172.1 ± 1.6 b	341.9 ± 2.4 c	61.0 ± 0.8 b	280.9 ± 2.8 c	153.1 ± 0.6 c
	*N*_180_	391.0 ± 2.0 a	87.0 ± 1.4 a	304.0 ± 1.7 a	176.8 ± 2.4 a	404.5 ± 3.4 a	69.2 ± 2.6 a	335.3 ± 0.8 a	180.0 ± 2.3 a	359.7 ± 3.5 a	66.7 ± 3.7 a	293.1 ± 1.6 a	176.8 ± 0.8 a
	*N*_360_	353.9 ± 2.4 b	85.0 ± 1.8 a	268.9 ± 1.6 b	163.8 ± 1.8 b	383.4 ± 3.3 b	64.4 ± 1.4 b	319.0 ± 1.7 b	173.1 ± 2.2 b	352.2 ± 3.0 b	61.0 ± 2.4 b	291.2 ± 0.7 b	156.7 ± 1.4 b
*D*_825_	*N*_0_	293.8 ± 1.3 c	61.3 ± 1.1 c	232.5 ± 1.7 c	138.2 ± 2.2 c	314.0 ± 2.2 c	58.3 ± 2.0 c	255.7 ± 1.8 c	131.0 ± 0.5 c	310.3 ± 2.4 c	54.3 ± 1.3 c	256.0 ± 1.2 c	137.4 ± 1.3 b
	*N*_180_	342.0 ± 1.7 a	72.0 ± 1.3 a	270.0 ± 2.0 a	157.0 ± 1.9 a	359.5 ± 1.6 a	63.6 ± 3.3 a	295.9 ± 1.7 a	162.1 ± 2.8 a	322.9 ± 2.2 a	62.4 ± 1.1 a	260.5 ± 1.4 a	153.9 ± 2.2 a
	*N*_360_	333.8 ± 1.2 b	69.1 ± 1.2 b	264.8 ± 2.3 b	152.6 ± 1.7 b	345.3 ± 1.1 b	61.9 ± 1.2 b	283.3 ± 2.0 b	149.6 ± 2.3 b	318.7 ± 3.7 b	60.7 ± 0.3 b	258.1 ± 2.0 b	138.8 ± 1.2 b
*D*_975_	*N*_0_	290.5 ± 2.1 c	59.7 ± 1.5 c	230.8 ± 1.8 c	136.6 ± 2.7 c	310.7 ± 1.6 c	56.7 ± 3.6 c	254.0 ± 3.6 c	129.3 ± 3.7 c	307.0 ± 2.4 c	52.7 ± 1.8 b	254.3 ± 2.6 b	135.7 ± 1.8 b
	*N*_180_	338.7 ± 1.6 a	70.4 ± 0.8 a	268.3 ± 2.6 a	155.3 ± 1.6 a	356.2 ± 2.7 a	62.0 ± 2.8 a	294.3 ± 2.7 a	160.4 ± 3.8 a	319.6 ± 3.1 a	60.8 ± 1.5 a	258.9 ± 2.7 a	152.3 ± 2.7 a
	*N*_360_	330.5 ± 2.2 b	67.4 ± 1.4 b	263.1 ± 2.5 b	150.9 ± 1.8 b	341.9 ± 3.0 b	60.3 ± 3.5 b	281.7 ± 3.3 b	147.9 ± 2.4 b	315.4 ± 1.6 b	59.0 ± 0.8 a	256.4 ± 1.1 ab	137.2 ± 1.4 b
*N*		**	**	**	**	**	**	**	**	**	**	**	**
*D*		**	**	**	**	**	**	**	**	**	**	**	**
*N* × *D*		**	*	**	**	**	**	**	**	**	**	**	**

* and ** indicate that the yield components are significantly influced by the N rate, plant density and their interactions at 0.05 and 0.01 levels, and ns indicates not significant. Different lowercase letters following the values in the same column indicate there is a significant difference in the same density level at P < 0.05.

### Distribution of vertical light and N and relationships with NUE

The canopy extinction coefficients for light (*K*_L_) and N (*K*_N_) were calculated using the cumulative LAI from the top of the canopy and the relative sunlight penetration. The *K*_L_ value from the U to M leaf layers decreased with higher N application rates. However, from the M to L leaf layers the *K*_L_ value was highest under the *N*_0_ input, then under the *N*_360_ input, with the minimum found for the *N*_180_ input (Table 3). Furthermore, *K*_L_ decreased at higher plant densities. As the SLN of the M leaf was the highest, the *K*_N_ from the U to M leaves had a negative value, while the *K*_N_ from the M to L leaves had a positive value. The value of *K*_N_ was the highest under the *N*_0_ input, followed by *N*_360_ input, with the minimum being under the *N*_180_ input.

**TABLE 3:** Influence of N rate and plant density on the canopy light extinction coefficient (*K*_L_, m^2^ ground m^–2^ leaf), canopy nitrogen extinction coefficient (*K*_N_, m^2^ ground m^–2^ leaf), and their ratio (*K*_N_*/K*_L_).

Density	N rate	Upper-middle			Middle-lower		
			
		*K*_L_	*K*_N_	*K*_N_ */K*_L_	*K*_L_	*K*_N_	*K*_N_ */K*_L_
**2019**							
*D*_525_	*N*_0_	0.31 ± 0.01 a	−0.05 ± 0.13 a	−0.15 ± 0.32 a	1.26 ± 0.11 a	0.37 ± 0.02 a	0.29 ± 0.01 a
	*N*_180_	0.28 ± 0.01 b	−0.02 ± 0.01 a	−0.07 ± 0.05 a	1.07 ± 0.11 b	0.20 ± 0.02 a	0.18 ± 0.03 b
	*N*_360_	0.26 ± 0.01 b	−0.04 ± 0.05 a	−0.16 ± 0.20 a	1.20 ± 0.05 ab	0.29 ± 0.24 a	0.24 ± 0.21 ab
*D*_675_	*N*_0_	0.25 ± 0.01 a	−0.04 ± 0.17 a	−0.18 ± 0.26 a	1.04 ± 0.09 a	0.29 ± 0.01 a	0.28 ± 0.01 a
	*N*_180_	0.23 ± 0.01 b	−0.01 ± 0.02 a	−0.05 ± 0.07 a	0.89 ± 0.11 b	0.15 ± 0.02 a	0.17 ± 0.03 b
	*N*_360_	0.21 ± 0.01 b	−0.03 ± 0.04 a	−0.16 ± 0.20 a	1.00 ± 0.05 ab	0.23 ± 0.18 a	0.24 ± 0.19 ab
*D*_825_	*N*_0_	0.28 ± 0.01 a	−0.35 ± 0.13 b	−1.30 ± 0.23 b	1.02 ± 0.03 a	0.61 ± 0.08 a	0.61 ± 0.09 a
	*N*_180_	0.23 ± 0.01 b	−0.12 ± 0.05 a	−0.53 ± 0.21 a	0.80 ± 0.04 c	0.17 ± 0.04 b	0.22 ± 0.05 b
	*N*_360_	0.24 ± 0.02 b	−0.14 ± 0.08 ab	−0.59 ± 0.28 a	0.91 ± 0.06 b	0.53 ± 0.19 a	0.59 ± 0.25 a
*D*_975_	*N*_0_	0.25 ± 0.00 a	−0.34 ± 0.11 b	−1.37 ± 0.28 b	0.93 ± 0.02 a	0.53 ± 0.08 a	0.58 ± 0.09 a
	*N*_180_	0.21 ± 0.01 b	−0.10 ± 0.04 a	−0.48 ± 0.19 a	0.75 ± 0.05 b	0.15 ± 0.06 b	0.20 ± 0.03b
	*N*_360_	0.20 ± 0.02 b	−0.12 ± 0.07 a	−0.59 ± 0.17 a	0.85 ± 0.08 ab	0.47 ± 0.14 a	0.57 ± 0.22 a
*N*		**	*	*	**	**	**
*D*		**	ns	ns	**	**	**
*N* × *D*		ns	ns	ns	ns	*	ns
**2020**							
*D*_525_	*N*_0_	0.22 ± 0.01 a	−0.37 ± 0.01 b	−1.67 ± 0.11 b	1.31 ± 0.09 ab	0.65 ± 0.03 a	0.50 ± 0.06 a
	*N*_180_	0.19 ± 0.00 b	−0.01 ± 0.02 a	−0.03 ± 0.10 a	1.20 ± 0.07 b	0.28 ± 0.01 c	0.23 ± 0.02 c
	*N*_360_	0.19 ± 0.01 b	−0.01 ± 0.07 a	−0.04 ± 0.23 a	1.37 ± 0.02 a	0.45 ± 0.07 b	0.33 ± 0.05 b
*D*_675_	*N*_0_	0.25 ± 0.00 a	−0.27 ± 0.24 b	−1.07 ± 1.33 b	1.22 ± 0.09 a	0.76 ± 0.40 a	0.62 ± 0.33 a
	*N*_180_	0.16 ± 0.01 b	−0.05 ± 0.03 ab	−0.33 ± 0.22 ab	1.02 ± 0.06 b	0.27 ± 0.18 b	0.27 ± 0.20 b
	*N*_360_	0.14 ± 0.02 b	0.01 ± 0.14 a	0.06 ± 0.53 a	1.15 ± 0.02 a	0.37 ± 0.05 ab	0.32 ± 0.04 ab
*D*_825_	*N*_0_	0.20 ± 0.00 a	−0.25 ± 0.13 b	−1.27 ± 0.64 b	0.92 ± 0.02 a	0.57 ± 0.07 a	0.61 ± 0.07 a
	*N*_180_	0.17 ± 0.01 b	−0.03 ± 0.05 a	−0.19 ± 0.29 a	0.68 ± 0.01 c	0.16 ± 0.11 b	0.23 ± 0.16 b
	*N*_360_	0.17 ± 0.01 b	−0.07 ± 0.23 a	−0.41 ± 0.87 a	0.77 ± 0.04 b	0.39 ± 0.04 a	0.50 ± 0.06 a
*D*_975_	*N*_0_	0.20 ± 0.00 a	−0.23 ± 0.14 b	−1.15 ± 0.69 b	0.88 ± 0.02 a	0.35 ± 0.21 a	0.38 ± 0.24 a
	*N*_180_	0.17 ± 0.01 b	−0.08 ± 0.09 a	−0.46 ± 0.53 a	0.70 ± 0.01 b	0.17 ± 0.12 b	0.24 ± 0.35 b
	*N*_360_	0.16 ± 0.00 b	−0.09 ± 0.26 a	−0.56 ± 0.40 a	0.78 ± 0.08 ab	0.28 ± 0.05 ab	0.35 ± 0.04 a
*N*		*	*	*	**	**	**
*D*		**	ns	ns	**	*	**
*N* × *D*		ns	ns	ns	ns	ns	ns

* and ** indicate that the yield components are significantly influced by the N rate, plant density and their interactions at 0.05 and 0.01 levels, and ns indicates not significant. Different lowercase letters following the values in the same column indicate there is a significant difference in the same density level at P < 0.05.

Parameter *K*_N_/*K*_L_ is an indicator of the N partitioning efficiency at the overall canopy level. This value from the U to M leaf positions was arranged in the order of *N*_180_ > *N*_360_ > *N*_0_, whereas the opposite tendency was found for the M to L leaf positions. It should be noted that the absolute *K*_N_/*K*_L_ value was higher at higher plant densities (*D*_825_ and *D*_975_) than that in the lower plant densities (*D*_525_ and *D*_675_).

**FIGURE 7:**
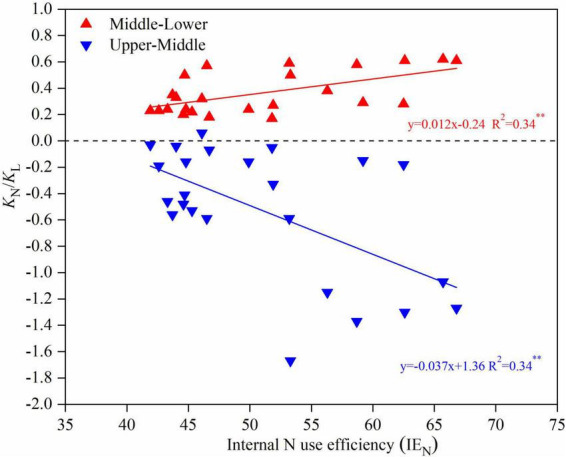
The N extinction coefficient with respect to light (*K*_N_/*K*_L_) vs. internal N use efficiency for upper-middle maize canopy (blue line) and middle-lower maize canopy (red line) in 2019 and 2020.

The relationship between *K*_N_/*K*_L_ and the internal N use efficiency (IE_N_) is shown in Figure 7. There was a significant positive correlation between the *K*_N_/*K*_L_ (from the M to L leaf positions) and IE_N_, where the fitted linear equation was *y* = 0.012*x* − 0.24, with a coefficient of determination of 0.34. There was a significant negative correlation between *K*_N_/*K*_L_ (from the U to M leaf positions) and IE_N_, where the fitted linear equation was *y* = −0.037*x* + 1.36 with a coefficient of determination of 0.34.

### Relationships between yield and NUE

**FIGURE 8:**
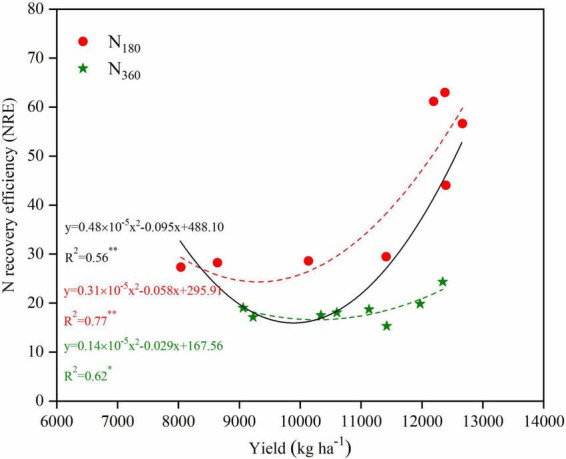
Relationships between yield and N recovery efficiency (NRE) for maize in 2019 and 2020. Non-linear regressions were fitted for overall data (black solid line), data for the *N*_180_ inputs (red dashed line) and *N*_360_ inputs (green dashed line).

There was a quadratic correlation between the yield and the N recovery efficiency (NRE) for the overall data, and this equation also fitted for the yield and NRE under *N*_180_ or *N*_360_ inputs with higher determination (Figure 8). The NRE under *N*_360_ inputs significantly lower than that under *N*_180_ input. At the same N level, the plant density management practices improved both the maize yield and NRE, particularly under the *N*_180_ inputs, and the NRE under the *D*_825_ treatment increased by 115.6% compared with the *D*_525_ treatment.

**FIGURE 9:**
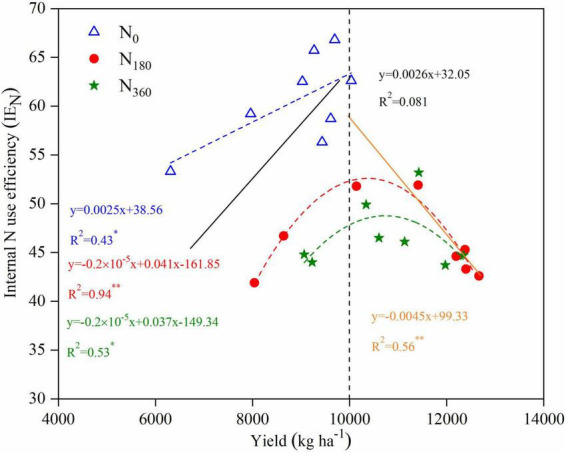
Relationships between yield and internal N use efficiency (IE_N_) for maize in 2019 and 2020. Linear regressions were fitted for the low yield data (<10,000 kg ha^– 1^; black solid line) and high yield data (≥10,000 kg ha^– 1^; orange solid line). Non-linear regressions were fitted for the data under the *N*_0_ inputs (blue dashed line), *N*_180_ inputs (red dashed line), and *N*_360_ inputs (green dashed line).

For a <10,000 kg ha^–1^ yield a positive linear correlation (*y* = 0.0026*x* + 32.05) appeared between the yield and IE_N_, whereas for a ≥10,000 kg ha^–1^ yield a significant negative linear correlation (*y* = −0.0045*x* + 99.33) appeared (Figure 9). At the same N levels, the fitted linear equation under *N*_0_ inputs was *y* = 0.0025*x* + 38.56 with a coefficient of determination of 0.43. The non-linear fitted equation under the *N*_180_ inputs was *y* = 0.2 × 10^–5^*x*^2^ + 0.041*x* − 161.85 with a coefficient of determination of 0.94, whereas under the *N*_360_ inputs it was *y* = 0.2 × 10^–5^*x*^2^ + 0.037*x* − 149.34 with a coefficient of determination of 0.53.

## Discussion

### Agronomic and photosynthetic attributes of canopy in high-yielding and high N efficiency maize

One of the most critical aspects of maize production is N nutrition. The application of N fertilizer within a certain range significantly increased maize yield (Zhao et al., 2018). In this study, N input increased the kernels per ear and grain weight of maize, particularly in terms of the number of kernels (Supplementary Table 1). Meanwhile, the NRE was reduced with higher N application rates (Figure 8), which suggests that the absorption of N by individual maize plants was limited; thus, it was necessary to increase the crop density. Currently, the density of summer maize in the North China Plain is ∼61,900 plants ha^–1^, which is much lower than that reported for high-yield maize in the United States (85,500–1,09,500 plants ha^–1^). Intensive planting has become a key measure and development trend toward achieving large-scale high-yielding maize on a global scale (Ma et al., 2020).

In this study, increasing plant density could further improve NRE under *N*_180_ inputs (Figure 8). This is because increasing the density accelerates the transfer of N nutrients from the stems and leaves to the grains (Lai et al., 2022). The highest NRE of maize in this experiment was 56.5–62.8% (2019–2020), while the average NRE of farmer practices in China was 25–30% (Li et al., 2019). Hence, increased plant density is important for reducing N fertilizer losses and environmental risks. Once the optimum plant density was exceeded, both the grain yields and NRE decreased (Figures 1, 8), whereas the risks of lodging, diseases, and insect pests increased (Xue et al., 2017).

In this study, the highest yield and NRE were observed for the *D*_825_/*N*_180_ treatment, but not for the *D*_975_ treatment (Figures 1, 8). Analysis of the agronomic and canopy attributes between different canopy layers for this treatment provided theoretical support for the development of maize populations with high yield and N efficiency in field production. Previous studies have demonstrated that LAI enhances light interception and further increase crop yield (Portes and Melo, 2014; Shi et al., 2016). In this study, the LAI of the *D*_825_/*N*_180_ treatment was substantially lower than that of the *D*_975_/*N*_180_ treatment, whereas it was markedly higher than that of the other N-density combinations (Table 1). Hence, LAI is not always accompanied by high yield (Remison and Lucas, 1982). Although LAI was not the highest in the *D*_825_/*N*_180_ treatment, it had the highest population PAR value (Figure 5). As N and plant density increased leaf area, the shading of the M and L leaves became even more severe under high N and planting density treatments. Insufficient light limits the physiological photosynthetic functions of leaves, even though it results in higher photosynthetic potential (Wang et al., 2017). This, suggests that population PAR is a better indicator of yield than leaf area or LAI.

Moreover, our findings revealed that the angles of the U leaves decreased more than those of the M and L leaves with increasing plant densities (Table 1). The substantial reduction in leaf area and angle of U-leaves seemed to favor more sunlight in the M and L leaf layers. Earlier studies proposed that the net photosynthetic rate of maize was greater in the mid-canopy than in the other leaves from the silking stage until physiological maturity (Dwyer and Stewart, 1986). In this study, the PI_total_ of the M leaves was the highest, followed by that of the U and L leaves (Figure 6). The low photosynthetic efficiencies in the L leaves were likely related to their long-term exposure to shade (Chen et al., 2015) but not to N nutrient concentration, as the lowest ABS/CS_0_ was recorded in the L leaves (Table 2 and Figure 6). Furthermore, the chlorophyll concentrations of the M leaves were the highest, followed by those of the L and U leaves. Meanwhile, the relative EC showed the opposite trend in the different leaf layers (Figure 6 and Supplementary Figure 2). Chlorophyll is often positively correlated with the functional periods of leaves, whereas the EC value is negatively correlated (Fu et al., 2020). These data indicate that the M leaves maintained a longer functional period than the L and U leaves. Furthermore, in this study, N deficits or excesses and increasing plant densities led to a significant reduction in whole-plant leaf photosynthetic efficiency (Figure 6 and Supplementary Figure 2). Thus, the selection of reasonable N-density treatments might initially impact leaf agronomic and physiological properties in different leaf layers, which are more conducive to the light-N matching of the vertical maize canopy.

### Allocation of vertical light and leaf N distribution in relation to grain yield and N use efficiency

Canopy productivity might be markedly improved by enhancing the light distribution to the leaf layer that contributes the most to grain yield. Several studies have demonstrated that the M leaves contributed the most, followed by the U and L leaves of maize (Dwyer and Stewart, 1986). This may be related to the photosynthetic products of M leaves following the principle of nearest distribution to preferentially supply the cobs (Yuan et al., 2015). Thus, it may be assumed that the higher the PAR is in M leaves, the more conducive the plant will be to high yields. In this study, the PAR value of M leaves in *D*_825_/*N*_180_ was the highest among all treatments; however, this was not only due to PAR ratio (Figure 5 and Supplementary Figure 1). Additionally, it is necessary to note that the *D*_825_/*N*_180_ treatment had the highest PAR ratio in the L leaves. Although the N concentration and photosynthetic capacity of L leaves were less than those of M and U leaves (Chen et al., 2015), they had sufficient leaf area (approximately 1.5 times higher than that of U leaves). Furthermore, the function of the L leaves is mainly to produce photosynthetic products to maintain the growth of roots and stems (Hao, 2017). Premature senescence of L leaves due to insufficient light accelerates root senescence, and absorption of mineral nutrients is restricted in the later growth stage (Borras et al., 2003). Then, to help the M and L leaves get more light, it is necessary to slow down the light reduction in the vertical direction, that is, a smaller *K*_L_ value. A smaller *K*_L_ value indicates a more uniform light distribution within the vertical canopy, which better matches the high-yielding canopy (Peng et al., 2008; Gu et al., 2017). Our results indicated that both the application of N and enhancing plant density could decrease the value of *K*_L_ (Table 3).

A linear, or more generally asymptotic relationship between photosynthesis and the leaf N content has been found in numerous studies (Xiong et al., 2015; Wu et al., 2019). Crop growth and production are dependent not only on the amount of total N absorbed by plants, but also on the vertical leaf N distribution within canopies (Li et al., 2013; Hikosaka, 2016). In many plant canopies, there is a vertical gradient of leaf N content per unit leaf area, which is higher in U-leaves (Huang et al., 2014; Niinemets et al., 2015). In this study, the N content of the M leaves was the highest, followed by that of the U and L leaves (Figure 6), which was distinct from that of rice and wheat (Shiratsuchi et al., 2006). Hence, the *K*_N_ from the upper-middle canopy had a negative value, whereas the *K*_N_ from the middle-lower canopy had a positive value. It was worth mentioning that the absolute value of *K*_N_ under moderate N inputs (*N*_180_) was lowest, and the high-yield treatment (*D*_825_/*N*_180_) had the lowest *K*_N_ (absolute value). This indicates that the N gradient distribution in the vertical canopy of high-yield maize was more uniform. Furthermore, the higher *K*_N_ under *N*_0_ input may have been related to low N availability. When the availability of N is low for plants, senescence of old leaves occurs; thus, the retranslocation of N from old to new leaves is accelerated, which also contributes to the high *K*_N_, as shown in Table 3.

It has been suggested that leaf N gradients observed in canopies represent a strategy for maximizing carbon assimilation (Hirose, 2005). In this study, a higher absolute *K*_N_/*K*_L_ value in the U-M canopy and a lower absolute *K*_N_/*K*_L_ value in the M-L canopy were observed in the *D*_825_/*N*_180_ treatment (Table 3). It can be used as an indicator for evaluating the quality of maize canopy structure in breeding and cultivation management. There was a significant negative linear correlation between the IE_N_ and grain yields when the yield was >10 t ha^–1^, whereas the NRE was significantly increased with enhanced yields (Figures 7, 8). Therefore, the mechanism of N-density matching to improve grain yields was not primarily determined by the retranslocation of N from the leaves to the grain, but rather by enhancing the absorption efficiency of N by roots. This viewpoint was similar to that of Yan et al. (2017) who suggested that planting density affected the ability of maize plants to use available soil N either insufficient or excessive density will result in a low NRE.

Previous studies have reported that insufficient plant densities and excessive use of N fertilizer were the main reasons for the lower corn yields and N use efficiencies in China (Meng et al., 2013). According to this study, increasing the plant density from 52,500 to 82,500 plants ha^–1^ improved both the grain yields and N use efficiency (NUE, including NRE and IE_N_). However, a further increase in plant density to 97,500 plants ha^–1^ induced grain yield losses and NUE reductions. In this study, the 82,500 plants ha^–1^ was similar to the optimal plant density (79,000 plants ha^–1^) in America (Ciampitti and Vyn, 2011), while it was lower than the 90,000–1,05,000 plant ha^–1^ for a superior high-yield study in China (Chen et al., 2011). It could not be ignored that the average N fertilizer inputs of super-high-yielding fields reached 774 kg N ha^–1^. Consequently, low NRE and substantial N losses have been reported in these fields, which is clearly not in line with the goals of modern crop nutrient management. Our study suggests that the potential negative effects of reduced N rate on yield attributes and grain yield can be compensated for increasing plant density to a certain range, and dense planting may be a feasible strategy to reduce N input in maize production.

## Conclusion

Increasing yields in conjunction with efficient N utilization is an important goal for sustainable agriculture. In this study, an N application rate of 180 kg ha^–1^ coupled with a plant density of 82,500 plants ha^–1^ achieved the highest yield and NRE, which resulted in higher plant densities and lower N inputs than traditional agricultural practices in Northern China. Moderate densification effectively reduced the leaf area and angles of the upper leaves while allowing more light to enter the middle and lower leaf layers, increasing the population TPAR. The N and chlorophyll concentrations, anti-aging capacity, and photosynthetic fluorescence efficacy of the middle leaves were much higher than those of other leaves. The lager leaf area compensated for the insufficient PAR absorption efficiency in the lower leaves. Thus, maintaining a certain light intensity in the lower leaf layer (indicated by a smaller *K*_L_) is key to achieving high yields. Furthermore, the reduced N rate without yield reductions under higher plant densities primarily attributed to the vertical N distribution, was more uniform.

## Data availability statement

The raw data supporting the conclusions of this article will be made available by the authors, without undue reservation.

## Author contributions

YY and YW conceived the idea and designed the study. JL, YZ, YH, PT, and YL conducted the experiments. YW and JL performed analyses and wrote the manuscript. All authors approved the final version of the manuscript.

## Abbreviations

N: nitrogen
PAR: photosynthetically active radiation
TPAR: total photosynthetically active radiation
LAI: leaf area index
*K*_L_: extinction coefficient for light
NRE: N recovery efficiency
IE_N_: internal N use efficiency
PI_*total*_: total performance index
*K*_N_: extinction coefficient for N
EC: relative leaf conductivity
M leaf: middle leaf
U leaf: upper leaf
L leaf: low leaf.
