# Supplementary material for: Nitrogen rates and plant density interactions enhance radiation interception, yield, and nitrogen use efficiencies of maize
Source: Front Plant Sci. 2022 Sep 23;13:974714. doi: 10.3389/fpls.2022.974714 (PMC9540852; doi:10.3389/fpls.2022.974714)
Supplement: Supplementary file 1 [file Data_Sheet_1.zip › Figure S1.docx]

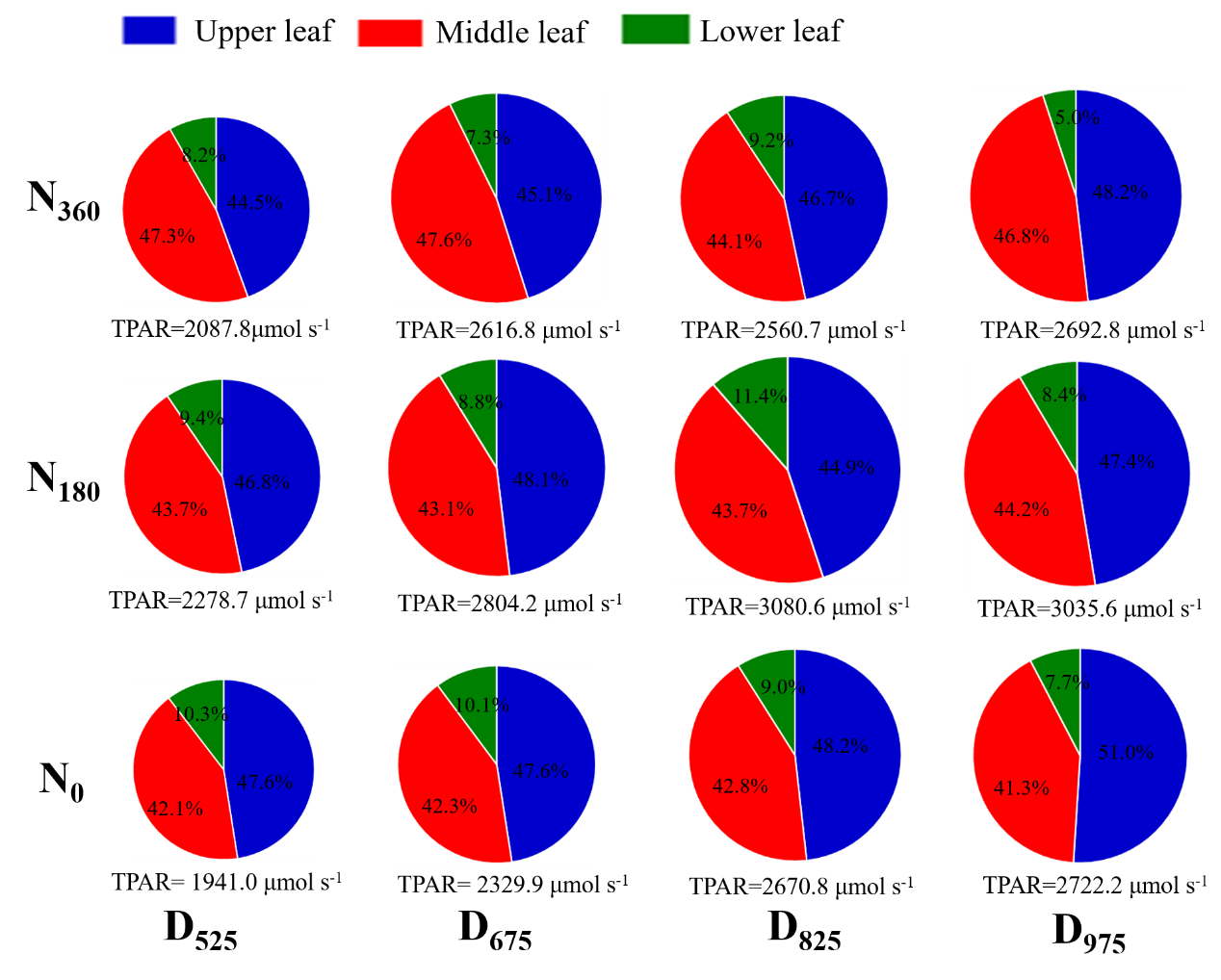


**Fig. S1.** Influence of N rate and plant density on the intercepted photosynthetically active radiation at different leaf positions in 2020. The size of pie diagram represents the TPAR of different leaf layers; the percentage of each section represents the TPAR ratio for the upper, middle, and lower leaf layers.
