# Supplementary material for: Nitrogen rates and plant density interactions enhance radiation interception, yield, and nitrogen use efficiencies of maize
Source: Front Plant Sci. 2022 Sep 23;13:974714. doi: 10.3389/fpls.2022.974714 (PMC9540852; doi:10.3389/fpls.2022.974714)
Supplement: Supplementary file 1 [file Data_Sheet_1.zip › Figure S2.docx]

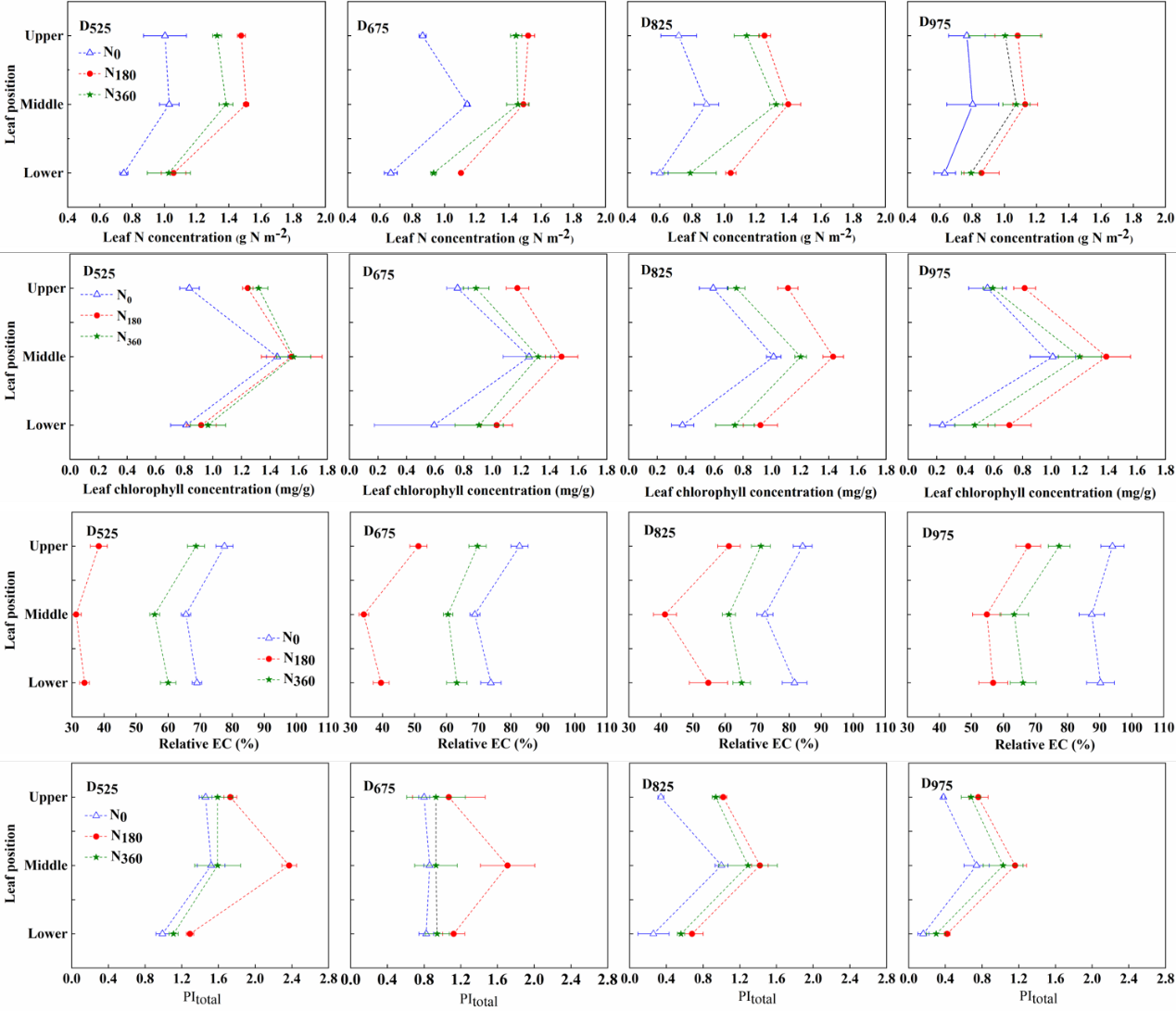


**Fig. S2.** Influence of N rate and plant density on the leaf N concentration, chlorophyll concentration, relative EC, and PI_total_ at different leaf positions in 2020.
