# Supplementary material for: Nitrogen rates and plant density interactions enhance radiation interception, yield, and nitrogen use efficiencies of maize
Source: Front Plant Sci. 2022 Sep 23;13:974714. doi: 10.3389/fpls.2022.974714 (PMC9540852; doi:10.3389/fpls.2022.974714)
Supplement: Supplementary file 1 [file Data_Sheet_1.zip › Table S1.docx]

**Table S1.** Influence of N rate and plant density on the yield components.

| Density | N rate | Kernels per ear (No.) | 100-grain weight (g) |
| --- | --- | --- | --- |
| 2019 |  |  |  |
| D_525_ | N_0_ | 574.3±7.4 b | 33.5±1.2 b |
|  | N_180_ | 594.8±21.2 ab | 35.8±0.9 a |
|  | N_360_ | 605.1±12.9 a | 34.1±0.9 ab |
| D_675_ | N_0_ | 507.9±18.6 a | 31.1±0.7 b |
|  | N_180_ | 509.1±4.1 a | 33.1±1.6 a |
|  | N_360_ | 512.8±11.1 a | 32.3±0.7 ab |
| D_825_ | N_0_ | 430.0±9.8 c | 29.5±1.4 b |
|  | N_180_ | 530.1±15.1 a | 32.2±0.9 a |
|  | N_360_ | 480.7±20.7 b | 30.4±0.6 ab |
| D_975_ | N_0_ | 405.9±14.7 a | 27.4±0.9 a |
|  | N_180_ | 417.1±16.5 a | 28.9±1.2 a |
|  | N_360_ | 409.5±17.1 a | 28.8±0.6 a |
| N |  | ** | ** |
| D |  | ** | ** |
| N×D |  | ** | ns |
| 2020 |  |  |  |
| D_525_ | N_0_ | 563.3±11.4 b | 32.9±1.1 b |
|  | N_180_ | 659.4±45.1 a | 35.5±1.2 a |
|  | N_360_ | 651.3±49.3 a | 34.5±2.5 ab |
| D_675_ | N_0_ | 459.2±11.1 b | 29.0±1.5 a |
|  | N_180_ | 613.2±38.5 a | 31.9±0.2 a |
|  | N_360_ | 626.9±25.4 a | 29.3±1.3 a |
| D_825_ | N_0_ | 533.3±17.4 a | 33.5±0.2 a |
|  | N_180_ | 546.5±40.9 a | 32.6±1.6 ab |
|  | N_360_ | 544.3±18.1 a | 31.1±0.3 b |
| D_975_ | N_0_ | 432.3±36.2 b | 30.8±1.8 a |
|  | N_180_ | 568.3±19.2 a | 27.8±0.3 b |
|  | N_360_ | 535.3±5.9 a | 27.2±1.9 b |
| N |  | ** | ** |
| D |  | ** | ** |
| N×D |  | ns | ** |

** and ** indicate that the yield components are significantly influenced by the N rate, plant density, and their interactions, at 0.05 and 0.01 levels, and ns indicates ‘not significant’ Different lowercase letters following the values in the same column indicate a significant difference at the same density level at P < 0.05.*
